# Temporal predictability promotes prosocial behavior in 5-year-old children

**DOI:** 10.1371/journal.pone.0217470

**Published:** 2019-05-28

**Authors:** Yingjia Wan, Hong Fu

**Affiliations:** School of Psychology, Nanjing Normal University, Nanjing, Jiangsu, China; Middlesex University, UNITED KINGDOM

## Abstract

Although interpersonal coordinative activities have been shown to produce prosocial effects in both adults and children, the underlying mechanisms remain unclear. While most approaches focus on the effect of mimicry and synchronous behavioral matching, we hypothesize that temporal predictability might play a central role in producing prosocial effects, as it directs coordination and might therefore strengthen shared intentionality. In a percussion task with pairs of 5-year old children, we manipulated temporal predictability and movement similarity/predictability between the pair’s movements. Temporal predictability was manipulated by instructing the pair to play the instruments either to beats that were evenly-spaced, and therefore predictable, or to beats that were random, and therefore unpredictable. Movement similarity/predictability was manipulated by having the pair play rhythmic patterns that were similar, predictable, or independent from each other. Children who played to predictable beats were more willing to solve problems cooperatively with their partners and to help when their partners had an accident. In contrast, there was no positive effect of rhythmic predictability or similarity. These results are the first to show that temporal predictability affects prosociality independent of movement similarity or predictability. We conclude that the predictable time frame commonly seen in coordinative activities may be key to strengthening shared intentionality and producing prosocial effects.

## Introduction

The ubiquity of cooperating, helping and sharing distinguishes human societies from those of other primates, and is arguably what makes human societies so successful in the animal world [1–4]. In recent years, understanding the origins and development of prosocial behavior has attracted widespread interest from a variety of fields including psychology [1–3], anthropology [5], economics [6,7], education [8] and public policy [9,10]. Elucidating the bases of prosocial behavior not only helps us better conceptualize ourselves as humans from an evolutionary perspective, but also provides important insights for understanding communication, interaction, collaboration and other aspects of social cognition [1,3,11]. Moreover, an emerging body of research on enhancing prosociality provides guidance for promoting cooperation in real-life circumstances and organizations, which is essential for addressing some of the most pressing issues in the modern society, including climate change, large scale conflicts, and inequality [3,4,12].

Prosocial behavior can be effectively enhanced by interpersonal synchrony [13–17]. In both adults and children, synchronous social activities such as singing, dancing and joint music making increase social bonding and facilitate subsequent cooperation and helping [15,18–23]. Infants as young as 14 months are more likely to engage in prosocial behaviors after being bounced in synchrony with the experimenter [24–26]. Similar effects are found with synchronous movement in non-musical contexts. In both adults and children, walking, tapping fingers, clapping hands and rocking chairs in synchrony have all been found to have affiliative effects, thereby enhancing prosociality [13,16,17,27]. Infants studies also show that synchronous movements in non-musical contexts increase helpfulness, although helping is more delayed than in musical contexts [28]. The positive social effects of interpersonal synchrony have also been found in naturalistic settings. Spontaneous synchrony in body movements during natural interactions is correlated with higher positive emotions, and it is likely that synchrony is playing a causal role [29].

A prevalent line of research uses the term synchrony to describe how individuals make the same movements at the same moment in time [13,17]. In these studies, interpersonal synchrony is typically manipulated by having groups of two or more people performing the same actions at the same time, such as tapping fingers to a beat [13], and results show that such synchronous rhythmic movements produce higher bonding effects than similar movements occurring at different tempos. These findings appear to suggest that “similar is better” for producing prosocial effects, as participants’ behaviors in the synchronous condition are always more similar, spatially and temporally, compared to the asynchronous condition. This argument is supported by an extensive body of research on prosocial effects of mimicry known as the “chameleon” effect [30–32].

Recent perspectives of interpersonal dynamics are challenging this predominant approach, arguing that the structure of interaction should be examined in broader contexts. For example, the interpersonal synergies perspective holds that interpersonal coordination should be viewed as a dynamic soft-ensemble system which minimizes variability in task-relevant movements but allows degrees of freedom to range freely in task-irrelevant areas [33]. Research guided by this framework has shown that increasing similarity is not necessarily beneficial to interpersonal interaction [34–36]. Instead, a mixture of similar and complementary behaviors is more beneficial for promoting collective interests and effective coordination [34,35]. This view also finds support in music studies. For instance, research on music improvisation found that performing freely to an open-ended backing track increased coordination and a sense of unity more so than playing under a predesigned structure [37]. Clearly, then, in order to further understand how synchrony occurs and influences social bonding, we need to expand the current standard paradigm of having participants perform highly similar, time-locked behaviors and begin to explore interactions with more variability and dynamics.

The interaction mode described in the interpersonal synergies perspective is common in real-life synchronous interactions. People frequently engage in activities where their actions are coordinated in time but their movements are different [38]. Examples include partner-dancing, percussion ensemble, and two people moving a piece of heavy furniture.

Recent studies have shown that simultaneous, complementary behaviors during interaction can produce positive social effects comparable to those of synchronous and identical behaviors. With 14-month infants, Cirelli et al. found that synchronous but anti-phase bouncing has prosocial effects that are comparable to in-phase bouncing [24]. Freestyle adult dancers who make synchronous but non-identical movements subsequently recall more information about each other than those dancing asynchronously [39]. Catmur and Heyes had participants perform hand or foot lifting actions while observing hand and foot lifting actions on a computer screen [40]. Increased feelings of closeness and helpfulness were comparable for contingent-but-different responses (e.g. hand lifting produced foot lifting, and foot lifting produced hand lifting) and contingent-and-similar responses (e.g. hand lifting produced hand lifting), compared to non-contingent responses. Taken together, these studies suggest that synchrony with some freedom in movements can have powerful bonding effects.

To the best of our knowledge, few studies have directly examined the element that is the most important to synchrony, namely *timing*. In contrast to mimicry and movement matching, synchrony stresses the timing, rather than the contents of the interactors’ behaviors [41]. The time interval between movements may play an important role in coordinating synchronous movements and is thus a plausible source of prosocial effects. Specifically, temporal predictability, that is the predictive relationship between the onset time of one participant’s movement and that of the other participant’s movement, might contribute to the prosocial effect of synchronous activities.

Although there is no direct evidence for this claim, past research provides some insights in support of our hypothesis. Specifically, temporal predictability guides behaviors in time-locked interpersonal coordinative activities, musical and non-musical [38], and therefore makes salient collective goals and shared intentionality which could increase prosociality [19]. Consider walking together. Without the aid of outside cues, coordinating one’s steps to walk side by side in perfect unison usually requires predicting when the other person will take a step. Another example is a jazz jam session in which each musician is playing different melodies on different beats. While the exact content and movement of each musician are hard to predict, everyone follows the same beat pattern. Temporal predictability, in this case dictated by a consistent beat, is the “common ground” that the musicians share, and it is crucial to maintaining a cohesive, interconnected ensemble.

Based on the infant and adult literature on the social effects of synchrony, together with the theory of shared intentionality and prosocial behavior, we hypothesized that temporal predictability plays an important role in producing prosocial effects in interpersonal coordinative activities. This question has previously been explored with infants experiencing passive movements [24]. In this study, Cirelli et al. found that being bounced in synchrony with the experimenter can increase prosociality in infants regardless of whether the movements were evenly spaced or following random beats. The infants were held by an adult bouncing to the beat instead of generating movements themselves. It is possible that older children engaging in active coordination will be more affected by the shared experience. It indeed would be particularly important to study the effects of interpersonal effects in kindergarten children. Nearly all children in China, and in many other countries, attend kindergarten, and one goal of kindergarten is to socialize children. Therefore, this is an ideal age group and environment where simple tasks that are empirically shown to increase prosocial behavior might eventually be incorporated into curricula. We conducted pilot study using kindergarten children, and found that five-year-old children are capable of learning to play instruments in response to visual guidance. We thus chose to study this age group and hypothesized that temporal predictability in active coordination would generate prosocial behavior as early as five years.

We designed a percussion game that guides children’s behaviors with visual stimuli presented on a computer screen and manipulated temporal predictability by adjusting beat predictability (evenly spaced beats vs. unevenly spaced beats). Five-year-old children, in same-sex dyads, played a duet: One child played the drum while the other played the bell, and they played in alternation as opposed to playing at the same time. By introducing this turn-taking dynamic where the participants’ movements are separated by a time interval, we were able to manipulate beat predictability without confounding it with simultaneous behavioral matching. We then measured children’s cooperative problem-solving behavior and spontaneous helping behavior in two subsequent tasks: an *apron-buttoning* task, which assessed cooperative problem solving; and a *beads* task adapted from Kirschner and Tomasello [19], which examined whether a child would help a partner who had an accident.

We also manipulated rhythmic similarity and predictability by having the pair play rhythmic patterns that were similar, predictable, or independent from each other. The prosocial effect of movement similarity is well-documented in the literature on both adults and children [30–32,42]. Recent adult studies have found that predictable but different movements can produce prosocial effects comparable to those of similar movements [40]. We thus predicted that rhythmic similarity and predictability in terms of relative movement patterns would produce prosocial effects when a temporal interval is added between the movements of the dyad.

## Methods

### Participants

This study involves 140 five-year-old children. The school and the parents provided written consent. The research meets all applicable standards with regard to the ethics of experimentation and research integrity. Ethical approval was obtained from the Ethics Committee of Nanjing Normal University. 16 children were excluded because they could not follow instructions either in the manipulation phase (n = 10) or in the test phase (n = 6). Excluding criteria are described in the results section. The final sample consisted of 124 children (74 boys and 50 girls, mean = 5 years and 6 months, range = 5.0 to 6.0 years).

Children were randomly paired with a familiar partner of the same gender from the same class and brought to a spare room in the kindergarten. In China, kindergarten children stay with the same class every day, so they know everyone in their classes. Therefore, the pairs were familiar with each other but not necessarily friends.

Gender differences and between-gender interactions were not the focus of this study. Therefore, we included only same-gender pairs and did not test additional participants to balance the number of boy and girl dyads. However, because there is a literature demonstrating that girls tend to score higher on tests of prosocial behavior [43–48], we included gender as a between-groups factor in the analyses.

### Study design

We used a between-participants, 2×3×2 design, with three independent variables (beat predictability, rhythm predictability, gender) and two dependent variables (cooperative problem solving, spontaneous helping).

### Procedure

#### Manipulation phase

**Fig 1:**
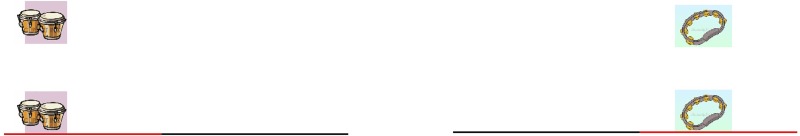
Visual stimuli of the percussion game. The left panel shows when the drum pattern hits the line, and the right panel shows when the bell pattern hits the line.

**Percussion game.** We developed a percussion game in which all the conditions are equally difficult, equally motivating and involve the same amount of movements and interactions. Post-hoc analysis reported in the results section confirmed that the conditions were of comparable difficulty. Two children were seated side by side and randomly assigned to a drum or a bell. The experimenters briefly introduced children to their instruments, and then told them that they would participate in a game. Children were presented with a computer screen, on which figures of drums and bells fell off from the top to the bottom one by one (Fig 1). About two-thirds of the way through the fall, the figure hit a line. When the figure hit the line, half of the line turned red, and the computer made a piano sound. Children were instructed to play their instruments whenever a figure representing their own instruments fell onto the line.

**Beat predictability manipulation**. Beat predictability was manipulated by assigning either a constant speed or a changing speed to the falling figures, thereby creating two conditions. In the *predictable beat* condition, the figures fell on evenly spaced beats at a constant speed (60 beats per minute, or bpm). In the *unpredictable beat* condition, the figures fell on unevenly spaced beats at a randomly changing speed (ranging from 45 bpm to 115 bpm).

**Rhythm predictability manipulation**. There are two rhythmic patterns in this game: one figure of the instrument fell off in one beat (the figure always fell on the downbeat), or two figures of the same instrument fell off in one beat (the first figure fell on the downbeat, and the second figure fell 0.5 second after the first figure, which was on the half beat in the predictable beat condition). We manipulated the relationship between the drum’s and the bell’s rhythmic patterns to create three conditions of rhythm predictability. In the *predictable*, *same rhythm* condition, the bell always repeated the drum’s rhythmic pattern (i.e. when one drum fell off, one bell fell off on the next beat; when two drums fell off, two bells fell off on the next beat). In the *predictable*, *different rhythm* condition, the bell’s rhythmic pattern was always different from the drum’s pattern, but the difference was predictable (i.e. when one drum fell off, two bells fell off on the next beat; when two drums fell off, one bell fell off on the next beat). In the *unpredictable*, *different rhythm* condition, the drum’s rhythmic pattern did not predict the bell’s rhythmic pattern (i.e. when one drum fell off, either one bell or two bells fell off on the next beat).

The beat and rhythm manipulations resulted in the following six conditions:

1. Predictable beat and predictable, same rhythm
  The figures fell off at a constant speed (60 bpm), and the bell always repeated the drum’s rhythmic pattern.
2. Predictable beat and predictable, different rhythm
  The figures fell off at a constant speed (60 bpm), and the bell’s rhythmic pattern was always different from the drum’s rhythmic pattern, but the difference was predictable.
3. Predictable beat and unpredictable, different rhythm
  The figures fell off at a constant speed (60 bpm), but the drum’s rhythmic pattern did not predict the bell’s rhythmic pattern.
4. Unpredictable beat and predictable, same rhythm
  Same as Condition 1, except that the figures fell off at a random speed (45–115 bpm).
5. Unpredictable beat and predictable, different rhythm
  Same as Condition 2, except that the figures fell off at a random speed (45–115 bpm).
6. Unpredictable beat and unpredictable, different rhythm
  Same as Condition 3, except that the figures fell off at a random speed (45–115 bpm).

Participants were randomly assigned to one of the six conditions. The experimenter and a research assistant first demonstrated the procedure for 30s, each performing one instrument according to the stimuli on the screen, and then let the children try to play along for 30s. Afterwards the children engaged in one round of semi-guided play and one round of unguided play, each lasting 1 min. In the semi-guided play, the experimenter and the research assistant played along with the children; in the unguided play, children played by themselves.

The percussion game was followed by one of the two evaluation tasks, the *apron task* and the *beads task*. After the first evaluation task, the pair were instructed to play the percussion game for another minute with no guidance to reinforce the manipulation. Then they proceeded to the second evaluation task.

#### Test phase

**Apron task.** The apron task examined whether children chose to solve problems cooperatively. The experimenter gave the children two aprons that had to be buttoned from behind. Then the experimenter and an assistant taught the dyad two ways of buttoning the apron: one was to do it by themselves, which took some time and effort; the other was to help each other, which was considerably easier and faster. The children each had a total of two buttons to fasten.

After making sure that both children had learned both ways, the experimenter told the children that she and the assistant had to leave the room briefly to make a phone call. When they came back, the children who had both of their buttons fastened would get a reward. The experimenter stressed that the children could choose to either button up themselves or help each other. She also emphasized that each child who completed the task would get the same reward. This was done to make sure that the children did not view the task as a competition. The experimenter and the assistant then left the room. They monitored the children through a hidden camera to ensure that both had completed the task. They then returned to the room and gave each child a candy bar.

**Beads task.** The beads task was adapted from Kirschner and Tomasello [19]. It examined whether a child chose to help a partner when the partner had a sudden accident.

**Fig 2:**
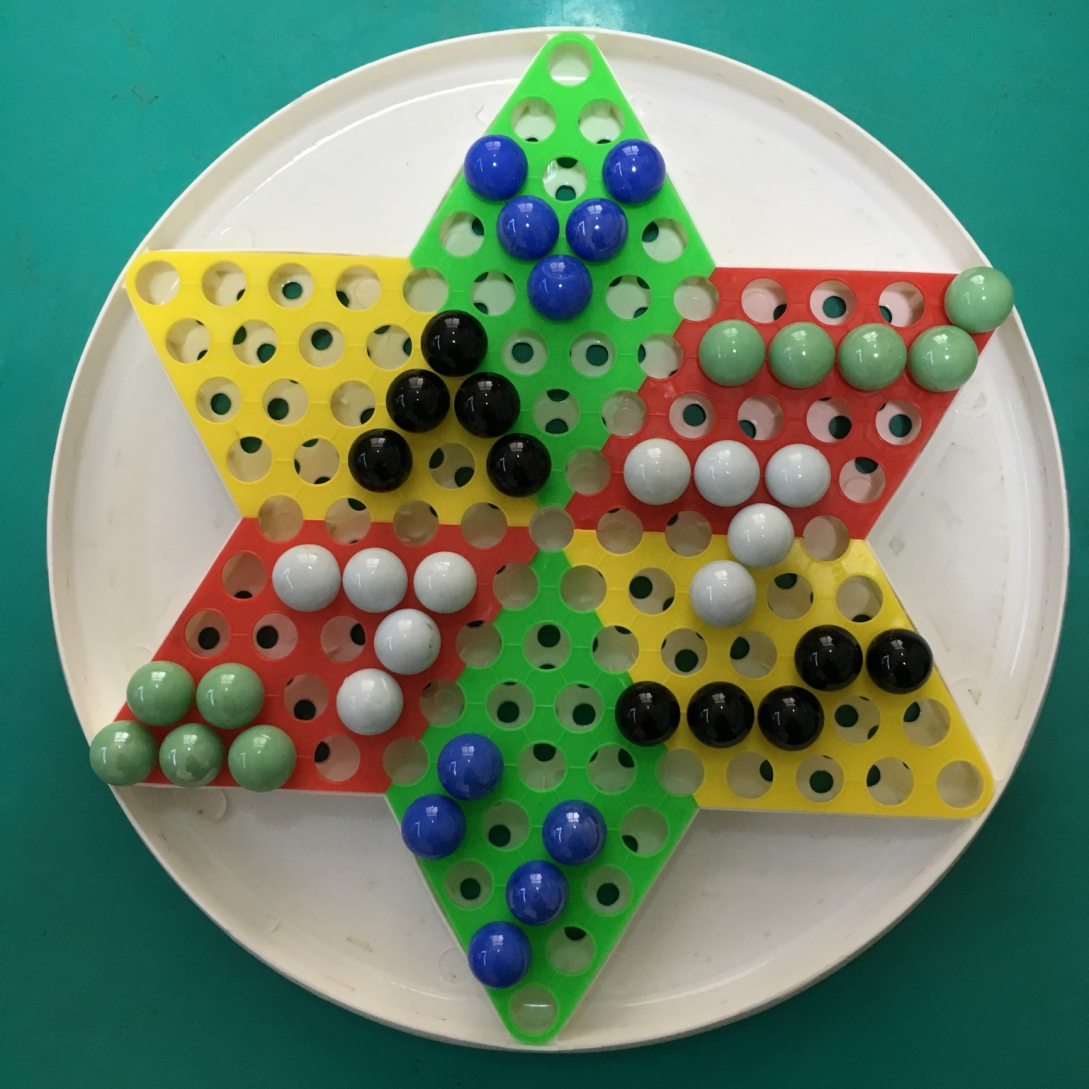
Model for the beads task.

**Cover story**. The experimenter presented the children with a box of white beads (10 beads in total) and a board, and told the children that their task was to arrange their beads according to a model (Fig 2). The experimenter and an assistant guided the children through arranging the white beads to make sure they understood the task. Children were instructed to build on the pattern they had already finished arranging and then arrange the rest of the beads following the model board. The experimenter placed the model board on a table in front of both children, and told the children that they were free to leave their seats to look at the board closely without touching it.

The children were told to finish the task color by color, in the order of black, blue and green. The experimenter then gave each child a box of black beads and placed the rest of the beads on the sides of the model (one box of blue beads and one box of green beads for each child on each side). The children were told that the experimenter and the assistant needed to leave the room briefly and that each child who finished the task before they returned would get a reward. The experimenter and the assistant then left the room. They returned after the dyad finished the tasks and gave each of them a cartoon eraser.

**Measurement.** One box of blue beads had an open bottom lid, so the beads would spill on the floor when it was picked up. The “victim” was either the child on the drum or the child on the bell. Assignment was random.

The session lasted around 20 minutes. The order of the two tasks, the apron task and the beads task, was counterbalanced across pairs and was balanced across trials. The experimenter (YW) conducted the experiment with the help of a research assistant blind to the hypotheses. All sessions were videotaped with a hidden camera.

## Results

### Apron task

YW and a research assistant blind to the hypotheses coded the video. We coded as “cooperation starts” when one child started helping the other child fasten a button. We coded the interaction between the dyad into one of four categories: (A) cooperation started before either child finished fastening the first button (n = 24), (B) cooperation started after one child or both children finished fastening the first button but before either finished fastening the second button (n = 10), (C) cooperation started after one child finished fastening the second button (n = 13), and (D) no cooperation (n = 15). Before conducting the primary analyses, we decided that because Category A and Category B both showed clear intention to solve the problem cooperatively, we would code both as “cooperative”. Inter-rater agreement (κ) was 0.98.

We performed a logistic regression analysis on cooperative behavior with beat predictability, rhythm predictability, gender and task order as fixed effects in the R environment. We included task order in the analysis to address the possibility that results in the second prosocial task could have been affected by prior experience with the first prosocial task and the additional round of percussion game. The results of this model are presented in Table 1. There were significant effects of beat predictability on cooperative behavior (β = 1.41, z = 2.30, p = 0.022, 95% CI [0.39, 2.88]). Children who played to predictable beats were more likely to cooperate with their partners in solving the apron task than children who played to unpredictable beats (69% vs. 42%). We also found a significant gender effect (β = 1.63, z = 2.59, p = 0.010. 95% CI [0.45, 2.96]), with boys acting more cooperatively than girls (68% vs. 36%). This is unexpected given previous literature [43–48]. We discuss this result later, presenting additional post-hoc analyses. We did not find any effects of rhythm predictability, order or interactions with these factors.

**Table 1:** Regression coefficients and test statistics from the logistic fixed-effects model on cooperative behavior.

	Beat Predictability	Rhythm (predictable/different)	Rhythm (predictable/similar)	Gender	Order
Est. β	1.41	0.97	0.12	1.63	0.98
z-value	2.30	1.35	0.16	2.59	1.61
p-value	0.022	0.176	0.873	0.010	0.108

We also performed two separate analyses that treated rhythmic patterns as a binary variable to further examine the effect of rhythmic predictability (predictable/same vs. predictable/different + unpredictable/different) and rhythmic similarity (predictable/same + predictable/different vs. unpredictable/different), and found similar results. At the suggestion of reviewers, we subsequently performed a set of fine-grained analyses that included the subcategories, and the results of the analyses fully complement the binomial analyses. The full results of these analyses are reported as Supporting Information (S1 File).

### Beads task

Coding was done from video by YW and a research assistant blind to the hypotheses. Two pairs were excluded from the final sample because the accident happened after the other child finished the task. Another pair was excluded because the “victim” left the room to seek help. We coded the reaction of “non-victim” to the accident into four categories: (A) immediately helped until the problem was solved (n = 22), (B) immediately helped but then left before the problem was completely solved (n = 3), (C) helped after finishing one’s own task or did not help but offered verbal encouragements or excuses (e.g. “Hurry up and pick them up!” “I will help you later!”) (n = 7), and (D) did not help or offer verbal encouragements/excuses (30). Since Category A and Category B both showed clear willingness to help others at one’s expense, we coded both as “helpful”. Inter-rater agreement (κ) was 0.95.

We performed a logistic regression analysis on helping behavior with beat predictability, rhythm predictability, gender and task order as fixed effects, and the results are presented in Table 2. There were significant effects of beat predictability on helping behavior (β = 1.38, z = 2.45, p = 0.014, 95% CI [0.31, 2.53]). Children who played to predictable beats were more likely to spontaneously help their partners in an accident than children who played to unpredictable beats (55% vs. 24%). We did not find any effect of rhythm predictability, gender, order or interaction.

**Table 2:** Regression coefficients and test statistics from the logistic fixed-effects model on helping behavior.

	Beat Predictability	Rhythm (predictable/different)	Rhythm (predictable/similar)	Gender	Order
Est. β	1.38	0.18	0.31	0.72	0.19
z-value	2.45	0.27	0.43	1.26	0.34
p-value	0.014	0.786	0.668	0.209	0.735

As with the apron task, we also performed a set of finer-grained analyses for the beads task, and the results of the analyses fully complement the binomial analyses. The full results of the analyses are reported in the Supporting Information (S1 File).

### Other variables

**Correlation between the apron task and the beads task.** A chi-squared test found no correlation between whether children cooperated in the apron task and whether children helped in the beads test (p = 0.933).

**Analyses of performance level in percussion game.** To evaluate the difficulty of the percussion game, the participants’ performance was coded from the video of the task. The level of each participant’s performance was rated by a music teacher blind to the hypothesis. The performance was rated as one of the following four levels: A (>80% accuracy), B (60%-80%), C (40%-60%), D (<40%). The participants’ performance level across conditions is presented in Table 3. An ordinal regression analysis found no effect of condition on performance level, nor was there any significant effect of rhythm predictability or beat predictability on performance level. We then ranked each dyad’s performance level based on the performance level of the individual participants: Very good (both participants were rated A, or one participant was rated A and the other was rated B), Good (both participants were rated B, or one participant was rated A and the other was rated C), Moderate (one participant was rated B and the other was rated C, or both participants were rated C), and Poor (at least one participant was rated D). If a participant was rated as “D”, the manipulation was considered invalid, and the dyad was removed from the final sample. Five pairs were removed for that reason. A logistic regression analysis on the final sample showed that there was no effect of performance level on either cooperation or helping behavior (ps > 0.5).

**Table 3:** Participants’ performance level across conditions.

	Condition						
	Predictable Beat			Unpredictable Beat			Sum
Performance Level	Predictable, Same Rhythm	Predictable, Different Rhythm	Unpredictable, Different Rhythm	Predictable, Same Rhythm	Predictable, Different Rhythm	Unpredictable, Different Rhythm	
A	16	16	13	16	17	15	93
B	4	5	7	6	5	6	33
C	2	1	1	1	2	2	9
D	2	0	1	1	0	1	5
Sum	24	22	22	24	24	24	140

**Further analyses of gender difference in apron task.** In the apron task, we found boys to be more cooperative than girls, which as noted was unexpected. A post-hoc explanation that occurred to us is that boys might have chosen to cooperate more because they were less confident in their button-skills. We reasoned that if that were the case, boys would take longer during the practice sections. In a post-hoc analysis, a research assistant blind to the hypothesis coded the amount of time each participant spent fastening the button of another person and the amount of time each participant spent learning to fasten his/her own buttons from behind (defined as the time length between the moment the participant grasps the button to the moment the button is fastened). Girls were faster than boys in both tasks (see Table 4). Linear regression analyses show that the differences are significant in both tasks (β = 4.44, z = 2.91, p = 0.004; β = 26.06, z = 3.08, p < 0.001). In addition, a linear regression analysis found a significant effect of time spent learning to fasten one’s own buttons on cooperating behavior (β = 0.01, z = 1.98, p = 0.047). Taken together these results suggest that the gender difference in cooperating behavior might be partially explained by differences in familiarity with the apron task.

**Table 4:** Time spent during practice session for the apron game (standard deviation in parentheses).

	Time spent fastening the button of another person (seconds)	Time spent learning to fasten one’s own buttons (seconds)
female	8.34(5.22)	50.48(24.16)
male	12.78(9.89)	76.54(45.91)

## Discussion

Playing percussion instruments to consistent, even-spaced beats increased prosocial behaviors in five-year-old children, regardless of whether the rhythmic patterns were similar to or predictive of one another. This indicates that temporal predictability might be a central contributor to prosocial effects of music activities and other similar forms of interpersonal coordination, beginning in early childhood.

One possible underlying mechanism is the predictive association between one’s action and that of others. Past studies have shown that the predictable relationship between one’s action and experienced events can increase feeling of closeness and prosociality in adults [40] and produce positive affect in infants [49]. In the current design, children playing to predictable beats could always predict the onsets of each other’s movements, and this predictivity alone could increase attention to each other and elicit feeling of connection. We acknowledge that this interpretation would be consistent with a perspective in which effects of synchrony or predictability arise from a Pavlovian process of mechanic association [40], and thus need not involve higher cognitive processes. However, an alternative explanation, which we favor, is based on shared intentionality, or “we” intentionality [14,19]. Sharing a predictable temporal frame leads participants to realize that they are involved in a task with a mutual goal, and this shared intentionality may cause them to become more helpful and cooperative in subsequent interactions. In the predictable beat condition, temporal predictability not only guides children to coordinate in time, but also integrates the movements of two children into a meaningful unit and acts as an important mutual framework for collaboration. From this perspective, children are not matching behaviors for the sake of being more similar, but rather for the purpose of fulfilling the mutual goal of co-performing a musical piece. In contrast, children in the unpredictable beat condition are not guided by a clear common cue, so they might not be as aware that they are either performing the same task or sharing a mutual goal. It will be important for future research to examine the degree of shared intentionality in collaborative activities, thus examining its effect on prosocial behavior and potentially adjudicating among these alternative classes of explanation. In addition, the effect of temporal predictability should also be explored in more naturalistic settings such as music ensemble, partner dancing and sports games. For example, during a joint task, success in predicting the onset of other’s behavior is likely to facilitate coordination, and this experience might thereby generate increased feelings of cohesion and prosocial tendencies.

Our findings demonstrating the importance temporal predictability differ from those of Cirelli et al. [24], who found no effect of temporal predictability in infants being bounced in response to music. However, the studies differ in important ways. Our participants actively make music and adjust their movements according to both the beat and the sounds produced by others. Therefore, predictable beat is more likely to have stronger influences on the participants’ experience and later interactions. It is also possible that older children are more sensitive to temporal predictability and the musicality of evenly-spaced movements, which then results in a developmental difference in the prosocial effects of beat predictability. It will be important for future studies to further explore these questions in a developmental context with children of different age groups.

We did not find any effect of rhythmic predictability or rhythmic similarity. While this is in line with the interpersonal synergies perspective which holds that similarity does not necessarily benefit interaction, it is somewhat surprising given the extensive literature that documents the prosocial effect of movement predictability and movement similarity [31,32,40,42]. However, most of that literature did not include temporal predictability as an independent variable, and thus the effect of temporal predictability might have been either neglected or confounded with other types of movement predictability. As for now, our explanation for the lack of results is that the effect of consistent beat might have overridden that of rhythm predictability. In the predictable beat condition, the steady temporal relationship between the dyad’s movements might be sufficient information for them to realize that they are performing the same task, so rhythmic similarity and predictability are not necessary elements for increasing feelings of cohesion and prosocial tendencies. In the unpredictable beat condition, the lack of predictable temporal relationship between the dyad’s movements might lead to low level of task-sharing and decreased interest in the partner’s behaviors, so the dyad might fail to notice that their rhythmic patterns are similar to each other or predictive of each other. Future studies could further explore the interaction between temporal predictability and movement predictability in variations of the present paradigm with more salient manipulation of the variables.

We also found boys to be more cooperative than girls in the apron task, which is surprising given the previous literature on gender differences which typically find high levels of prosociality for girls and occasionally no gender differences. Based on the analysis of task learning time, we concluded that the gender difference in cooperating behavior in the current study is likely caused by the difference in familiarity with the task. However, there might also be a cultural explanation for the gender difference. Since most of the studies that found girls to be more prosocial are conducted in Western cultures, it is possible that the gender pattern might be mitigated or even reversed in China. In fact, a recent study in our lab found that 4-year-old boys were more generous than girls in a dictator task, which we attributed to the emphasis that Chinese culture places on the importance of generosity for males [50]. In future studies it will be important to use cross-cultural designs to further explore the effect of culture on interpersonal coordination and prosocial development.

In conclusion, our study is the first to demonstrate that temporal predictability between a dyad’s movements produces prosocial effects in five-year-old children and that temporal predictability affects prosociality independent of similarity or predictability of the relative movement patterns. Temporal predictability is universally present in time-locked joint actions and characteristic of coordinated social behaviors that are known to increase mutual bonding and prosocial tendencies, such as joint music making and synchrony [13–29]. Therefore, our results suggest that the prosocial effects of interpersonal coordination may be modulated by the consistency and predictability of temporal frames.

## Supporting information

S1 File Detailed analyses of the apron task and the beads task. (DOCX)

S1 Video Demonstration video of the percussion game. (MP4)
