# Supplementary material for: Temporal predictability promotes prosocial behavior in 5-year-old children
Source: PLoS One. 2019 May 28;14(5):e0217470. doi: 10.1371/journal.pone.0217470 (PMC6538183; doi:10.1371/journal.pone.0217470)
Supplement: S1 File — (DOCX) [file pone.0217470.s001.docx]

**S1 File. Detailed Analyses of the Apron Task and the Beads Task.**

1. **Analyses of rhythm predictability and rhythm similarity**

**Analysis of rhythm predictability for the apron task.** In this analysis, we treated rhythmic patterns as a binary variable to further examine the effect of rhythmic predictability (predictable/same vs. predictable/different + unpredictable/different). We performed a logistic regression analysis on cooperative behavior with beat predictability, rhythm predictability (predictable/same vs. predictable/different + unpredictable/different), gender and task order as fixed effects in R environment. The results of this model are presented in Table 1.

**Table 1. Regression coefficients and test statistics from the logistic fixed-effects model on cooperative behavior (predictable rhythm vs. unpredictable rhythm).**

|  | Beat Predictability | Rhythm Predictability | Gender | Order |
| --- | --- | --- | --- | --- |
| Est. β | 1.37 | 0.58 | 1.57 | 0.93 |
| z-value | 2.27 | 0.93 | 2.54 | 1.56 |
| p-value | 0.023* | 0.355 | 0.011* | 0.120 |

**Analysis of rhythm similarity for the apron task.** In this analysis, we treated rhythmic patterns as a binary variable to further examine the effect of rhythmic similarity (predictable/same + predictable/different vs. unpredictable/different). We performed a logistic regression analysis on cooperative behavior with beat predictability, rhythm similarity (predictable/same + predictable/different vs. unpredictable/different), gender and task order as fixed effects in R environment. The results of this model are presented in Table 2.

**Table 2. Regression coefficients and test statistics from the logistic fixed-effects model on cooperative behavior (same rhythm vs. different rhythm).**

|  | Beat Predictability | Rhythm Similarity | Gender | Order |
| --- | --- | --- | --- | --- |
| Est. β | 1.34 | 0.41 | 1.66 | 0.92 |
| z-value | 2.23 | 0.63 | 2.68 | 1.55 |
| p-value | 0.026* | 0.518 | 0.007* | 0.121 |

**Analysis of rhythm predictability for the beads task.** In this analysis, we treated rhythmic patterns as a binary variable to further examine the effect of rhythmic predictability (predictable/same vs. predictable/different + unpredictable/different). We performed a logistic regression analysis on helping behavior with beat predictability, rhythm predictability (predictable/same vs. predictable/different + unpredictable/different), gender and task order as fixed effects in R environment. The results of this model are presented in Table 3.

**Table 3. Regression coefficients and test statistics from the logistic fixed-effects model on helping behavior (predictable rhythm vs. unpredictable rhythm).**

|  | Beat Predictability | Rhythm Predictability | Gender | Order |
| --- | --- | --- | --- | --- |
| Est. β | 1.38 | 0.24 | 0.72 | 0.19 |
| z-value | 2.45 | 0.40 | 1.25 | 0.34 |
| p-value | 0.014* | 0.693 | 0.211 | 0.732 |

**Analysis of rhythm similarity for the beads task.** In this analysis, we treated rhythmic patterns as a binary variable to further examine the effect of rhythmic similarity (predictable/same + predictable/different vs. unpredictable/different). We performed a logistic regression analysis on helping behavior with beat predictability, rhythm similarity (predictable/same + predictable/different vs. unpredictable/different), gender and task order as fixed effects in R environment. The results of this model are presented in Table 4.

**Table 4. Regression coefficients and test statistics from the logistic fixed-effects model on helping behavior (same rhythm vs. different rhythm).**

|  | Beat Predictability | Rhythm Similarity | Gender | Order |
| --- | --- | --- | --- | --- |
| Est. β | 1.38 | 0.21 | 0.70 | 0.19 |
| z-value | 2.45 | 0.61 | 1.23 | 0.34 |
| p-value | 0.014* | 0.564 | 0.217 | 0.734 |

1. **Categorical analyses of the apron task and the beads task**

**Categorical analyses of the apron task.** As stated in the result section, we coded the interaction between the dyad into one of four categories: (A) cooperation started before either child finished fastening the first button (n = 24), (B) cooperation started after one child or both children finished fastening the first button but before either finished fastening the second button (n = 10), (C) cooperation started after one child finished fastening the second button (n =13), and (D) no cooperation (n = 15). The categories were ranked according to the level of cooperativeness: very high (A), high (B), intermediate (C) and low (D). We performed an ordinal regression analysis on cooperative categories using the MASS package in R environment, and the results are presented in Table 5. Children who played to predictable beats were more cooperative than children who played to unpredictable beats (*OR* = 1.25, t = 2.47, p = 0.014, 95% CI [0.28, 2.27]), and boys were more cooperative than girls (*OR* = 1.46, t = 2.80, p = 0.005, 95% CI [0.46, 2.51]). There were no effect of rhythm predictability, order or interaction.

**Table 5. Regression coefficients and test statistics from the ordinal logistic model on cooperative categories.**

|  | Beat Predictability | Rhythm (predictable/different) | Rhythm (predictable/similar) | Gender | Order |
| --- | --- | --- | --- | --- | --- |
| Est. β | 1.25 | 0.36 | 0.32 | 1.46 | 0.68 |
| t-value | 2.47 | 0.59 | 0.52 | 2.80 | 1.35 |
| p-value | 0.014* | 0.553 | 0.602 | 0.005* | 0.176 |

**Categorical analyses of the beads task.** As described in the text, we coded the reaction of “non-victim” to the accident into four categories: (A) immediately helped until the problem was solved (n =22), (B) immediately helped but then left before the problem was finished (n = 3), (C) helped after finishing one’s own task or did not help but offered verbal encouragements or excuses (e.g. “Hurry up and pick them up!” “I will help you later”) (n = 7), and (D) did not help or offer verbal encouragements/excuses (30). The categories were ranked according to the efforts made to help pick up beads: very high (A), high (B), intermediate (C) and low (D). We performed an ordinal regression analysis on helping categories, and the results are presented in Table 6. Children who played to predictable beats made more efforts to help their partners solve problems than children who played to unpredictable beats (*OR* = 1.05, t = 2.06, p = 0.40, 95% CI [0.07, 2.08]). There were no effect of rhythm predictability, order or interaction.

**Table 6. Regression coefficients and test statistics from the ordinal logistic model on helping categories.**

|  | Beat Predictability | Rhythm (predictable/different) | Rhythm (predictable/similar) | Gender | Order |
| --- | --- | --- | --- | --- | --- |
| Est. β | 1.05 | 0.42 | 0.40 | 0.85 | 0.66 |
| t-value | 2.06 | 0.69 | 0.63 | 1.63 | 1.27 |
| p-value | 0.040* | 0.488 | 0.528 | 0.103 | 0.202 |

**Correlation between the apron task and the beads task.** A Kendall tau test found no correlation between level of cooperation and level of helpfulness (p = 0.884).
